# A Systematic Review of Functional Outcomes Following Surgical and Conservative Management of Congenital Radioulnar Synostosis

**DOI:** 10.7759/cureus.105153

**Published:** 2026-03-13

**Authors:** Callum Counihan, William Kirk, Valentina Palloni, Emma Stewart, Neil Segaren, Kunalan Maruthainar

**Affiliations:** 1 Trauma and Orthopaedics, Epsom and St. Helier University Hospitals NHS Trust, London, GBR; 2 Physical Medicine and Rehabilitation, University of Milan, Milan, ITA; 3 Trauma and Orthopaedics, Frimley Health NHS Foundation Trust, Frimley, GBR; 4 Trauma and Orthopaedics, Royal National Orthopaedic Hospital, London, GBR

**Keywords:** congenital radioulnar synostosis, derotation osteotomy, forearm surgery, paediatric orthopaedics, surgical outcomes research

## Abstract

Congenital radioulnar synostosis (CRUS) is a rare upper limb malformation causing fixed forearm malposition. Management remains controversial, with no consensus on indications for surgery or the optimal operative technique. This systematic review evaluated reported outcomes of surgical and conservative management of CRUS.

A systematic review was conducted in accordance with PRISMA and registered with PROSPERO (CRD42019148014). MEDLINE, Embase, Journals@Ovid, Cochrane, CINAHL, BMJ Case Reports, and Google Scholar were searched to 20 February 2026. Studies of any design reporting management of CRUS were included. Data were extracted on laterality, treatment type, forearm position, functional outcomes, complications, and follow-up. Risk of bias was assessed using Joanna Briggs Institute (JBI) critical appraisal tools.

A total of 55 studies were included, comprising 662 patients and 856 forearms; 195 patients (29.5%) had bilateral involvement. Surgical management was reported in 552 patients and conservative management in 110. There was no consistent indication for surgery: some studies used functional limitation while others used fixed pronation thresholds. Surgically treated patients had greater deformity and were more likely to have bilateral disease than those managed non-operatively. Derotational procedures consistently improved resting forearm position, achieving a mean postoperative position of 1° pronation, and were associated with improved functional outcomes. In contrast, simple synostosis resection generally failed to maintain motion because of recurrence, while more complex interposition procedures showed more promising but less reproducible results. Complications were reported in 61 surgically managed patients (11.1%), including nerve palsy, recurrence, loss of alignment, vascular compromise, non-union/delayed union, wound infection, and compartment syndrome.

Evidence for CRUS management is limited by low quality, heterogeneous studies. Operative treatment may improve function but carries a meaningful risk of complications. Treatment should therefore be individualised, with decisions based on functional limitation, compensatory shoulder and elbow motion, deformity severity, and bilateral involvement.

## Introduction and background

Congenital radioulnar synostosis (CRUS) is an infrequent malformation in which there is an abnormal connection between the radius and the ulna due to an embryological failure of separation. CRUS is thought to be caused by persistence of the cartilaginous anlage between the radius and ulna during the seventh week of development, resulting in a bony or fibrous synostosis. As the forearm is positioned in pronation in utero this typically results in varying degrees of fixed forearm pronation, although fixed supination can occur [1,2]. 

Cleary and Omer proposed a radiological classification for CRUS. Type one shows no radiographic abnormality, type two demonstrates a bony bridge, and types three and four denote synostosis with posterior and anterior radial head dislocations respectively [1]. Type three is the most frequently reported variant [1,3,4]. 

Most reported cases of CRUS do not have associated developmental abnormalities. However, a range of associated developmental abnormalities have been reported including: talipes equinovarus, absent thumb, coalescence of the carpal bones, symphalangism, bowing of the radius, acropolysyndactyly, arthrogryposis, mandibulofacial dysostosis, renal hypoplasia, partial carpal coalition and hypoplasia of the thoracic cage [3,5]⁠. CRUS has also been reported in combination with genetic conditions such as Apert and Klinefelter syndromes [3,5]⁠. The majority of reported CRUS cases are sporadic in nature however familial association has been reported in some cases [6,7].

The impact of CRUS on patients is variable. Mild cases may have limited functional restrictions and remain undiagnosed as patients are able to compensate well. This can be due to wrist hypermobility, an incomplete synostosis resulting in some limited pronation-supination or a combination of the two [3,8,9]. However, bilateral cases and those with a significant fixed pronation deformity may experience substantial functional limitations [1]. In practice, this is most apparent in activities that require forearm rotation and palm orientation such as personal hygiene, dressing, eating with utensils, and certain sporting or school tasks [1].

At present there are multiple controversies surrounding management and no clear consensus on indications for surgery, the optimal operative procedure or even goals of treatment. This systematic review aimed to evaluate reported outcomes of surgical and conservative management of CRUS, with particular focus on indications for intervention, forearm position, functional outcomes, and complications.

## Review

Methods

*Search Strategy* 

A systematic review was conducted in accordance with the Preferred Reporting Items for Systematic Reviews and Meta-Analyses (PRISMA) guidelines and registered with PROSPERO (CRD42019148014). Electronic database searches were performed on 20 February 2026 using the Ovid platform in Ovid MEDLINE(R) ALL (1946 to 20 February 2026), Embase (1974 to 17 February 2026), and Journals@Ovid Full Text (20 February 2026). Search terms for CRUS (including relevant related terms) were used, with English-language and human limits applied where available. Additional searches were undertaken in the Cochrane Library, BMJ Case Reports, CINAHL, and Google Scholar, and the reference lists of included studies were reviewed to identify further relevant articles.

Eligibility Criteria and Identification

This systematic review included studies of any design reporting on the management of CRUS. Exclusion criteria were: 1) studies not related to the management of CRUS, 2) papers written in languages other than English, and 3) animal studies.

Electronic searches were carried out by CC and WK with the assistance of a health service librarian. Title and abstract screening to identify potentially eligible papers was then carried out by CC, NS, and KM to ensure consensus.

Data Extraction and Critical Appraisal

Data were extracted by CC and VP using a data collection table. A selection of these data was checked by NS and KM.

The extracted data included age, laterality of synostosis, type of management (surgical or conservative), surgical procedure type, complications, length of follow-up, and functional outcomes. Data extraction was deemed complete once all reviewers agreed.

The methodological quality of the included studies was assessed using the Joanna Briggs Institute (JBI) critical appraisal tools, selected according to study design: the JBI Critical Appraisal Checklist for Case Series (10 items) for case series [10], and the JBI Critical Appraisal Checklist for Case Reports (8 items) for case reports [10]. Each study was appraised independently against each item and rated as Yes, No, or Not applicable. To provide an overall per-study rating, we summed the number of items rated “Yes” and categorised the overall risk of bias as follows: for case series, low (8-10 “Yes”), moderate (5-7 “Yes”), or high (0-4 “Yes”); for case reports, low (6-8 “Yes”), moderate (4-5 “Yes”), or high (0-3 “Yes”).

Given the limited availability of high-quality studies and the heterogeneous nature of the interventions and outcome measures, a narrative synthesis was undertaken.

Results 

**Figure 1:**
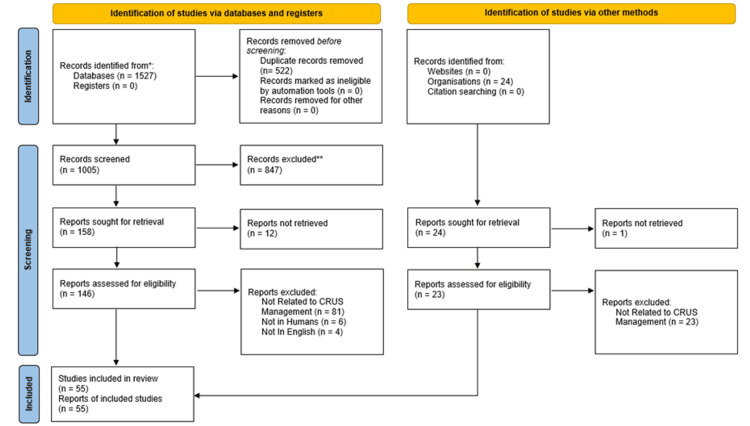
PRISMA flow diagram of study selection. PRISMA: Preferred Reporting Items for Systematic Reviews and Meta-Analyses.

The initial literature search resulted in a total of 1,527 papers. Once duplicates were removed, 1,005 records remained. After an initial screening of titles and abstracts, a further 847 were excluded, and 158 papers were sought for retrieval. Of these, 12 could not be retrieved, leaving 146 available for review. From these, 55 eligible papers were identified for inclusion. Figure 1 shows full details of study selection.

These included 662 patients and 856 forearms, of which 195 were bilateral (29.5%). Surgical management was reported in 552 patients, and conservative management was reported in 110 patients. Table 1 presents full details of the included studies.

**Table 1:** Characteristics of the included studies. CRUS: Congenital radioulnar synostosis; ROM: Range of motion; QuickDASH: Quick Disabilities of the Arm, Shoulder and Hand; Jebsen-Taylor: Jebsen-Taylor Hand Function Test.

Reference (author, year)	Study design	Sample size	Study population	Key findings
Zarantonello P et al. (2025) [11]	Case series	97 patients / 122 forearms	CRUS; 52 surgical and 45 non-operative cases	Procedure: Osteotomy (various). Forearm position: 69.1° pronation preoperatively to 2.6° pronation postoperatively (15.1° pronation in the non-operative group). Functional outcome: QuickDASH. Complications: 6.
Dong Y et al. (2024) [12]	Case series	6 patients / 9 forearms	CRUS; 6 surgical and 0 non-operative cases	Procedure: Osteotomy of synostosis with local fat flap interposition. Forearm position: 65.0° pronation preoperatively. Postoperative ROM: 85°. Functional outcome: Failla. Complications: 1.
Luo X et al. (2024) [13]	Case series	22 patients / 24 forearms	CRUS; 22 surgical and 0 non-operative cases	Procedure: Osteotomy of the ulna and radius. Forearm position: 75.0° pronation preoperatively to 3.8° pronation postoperatively. Functional outcome: Failla. Complications: 1.
Kanaya F et al. (2023) [14]	Case series	25 patients / 26 forearms	CRUS; 25 surgical and 0 non-operative cases	Procedure: Osteotomy and interposition of a fascial graft. Forearm position: 34.8° pronation preoperatively. Postoperative ROM: 86.5°. Functional outcome: Not reported. Complications: Not reported.
Gandhi S et al. (2023) [15]	Case series	6 patients / 7 forearms	CRUS; 6 surgical and 0 non-operative cases	Procedure: Osteotomy of the ulna and radius. Forearm position: 71.5° pronation preoperatively to 7.5° pronation postoperatively. Functional outcome: Failla. Complications: Not reported.
Bo H et al. (2023) [16]	Case series	17 patients / 26 forearms	CRUS; 17 surgical and 0 non-operative cases	Procedure: Osteotomy of the ulna and radius. Forearm position: 71.4° pronation preoperatively to 7.3° pronation postoperatively. Functional outcome: Not reported. Complications: Not reported.
Tan W et al. (2022) [17]	Case series	27 patients / 39 forearms	CRUS; 27 surgical and 0 non-operative cases	Procedure: Osteotomy of synostosis. Forearm position: 59.7° pronation preoperatively to 8.59° pronation postoperatively. Functional outcome: Failla. Complications: 1.
Martínez-Álvarez S et al. (2022) [18]	Case series	14 patients / 18 forearms	CRUS; 14 surgical and 0 non-operative cases	Procedure: Osteotomy of the ulna and radius. Forearm position: 80.0° pronation preoperatively to 0° postoperatively. Functional outcome: Not reported. Complications: 0.
Bai F et al. (2022) [19]	Case series	11 patients / 16 forearms	CRUS; 11 surgical and 0 non-operative cases	Procedure: Osteotomy and interposition of a free flap. Forearm position: 67.3° pronation preoperatively. Postoperative ROM: 71.0°. Functional outcome: Not reported. Complications: 4.
Hamiti Y et al. (2022) [20]	Case series	10 patients / 12 forearms	CRUS; 10 surgical and 0 non-operative cases	Procedure: Osteotomy of the ulna and radius. Forearm position: 56.7° pronation preoperatively to 3.3° pronation postoperatively. Functional outcome: Failla. Complications: 0.
Zhang ZQ et al. (2021) [21]	Case series	4 patients / 5 forearms	CRUS; 4 surgical and 0 non-operative cases	Procedure: Osteotomy of synostosis. Forearm position: 98.0° pronation preoperatively to 38.0° pronation postoperatively. Functional outcome: Not reported. Complications: 0.
Kepenek-Varol B and Hoşbay Z (2020) [22]	Case report	1 patient / 2 forearms	CRUS; 0 surgical and 1 non-operative case	Procedure: Non-operative. Forearm position: Not reported. Functional outcome: Not reported. Complications: Not reported.
Jia Y et al. (2020) [23]	Case report	1 patient / 1 forearm	CRUS; 1 surgical and 0 non-operative cases	Procedure: Synostosis osteotomy with interposition of a fascial graft. Forearm position: Not reported. Functional outcome: Not reported. Complications: 1.
Pei X and Han J(2019) [24]	Case series	31 patients / 36 forearms	CRUS; 31 surgical and 0 non-operative cases	Procedure: Synostosis osteotomy. Forearm position: 62.0° pronation preoperatively to 7.5° pronation postoperatively. Functional outcome: Failla. Complications: 3.
Barrera-Ochoa S et al. (2019) [25]	Case report	1 patient / 2 forearms	CRUS; 1 surgical and 0 non-operative cases	Procedure: Osteotomy and interposition of a silicone implant. Postoperative ROM: 100.0°. Functional outcome: Not reported. Complications: 0.
Satake H et al. (2018) [26]	Case series	9 patients / 12 forearms	CRUS; 9 surgical and 0 non-operative cases	Procedure: Osteotomy of the mid-radius. Forearm position: 51.0° pronation preoperatively to 4.0° supination postoperatively. Functional outcome: QuickDASH. Complications: 0.
Natwa N et al. (2018) [8]	Case report	1 patient / 2 forearms	CRUS; 0 surgical and 1 non-operative case	Procedure: Non-operative. Forearm position: Not reported. Functional outcome: Not reported. Complications: 0.
Tsai J (2017) [27]	Case report	1 patient / 2 forearms	CRUS; 0 surgical and 1 non-operative case	Procedure: Not reported. Forearm position: Not reported. Functional outcome: Not reported. Complications: 0.
Kanaya K et al. (2016) [28]	Case series	4 patients / 6 forearms	CRUS; 4 surgical and 0 non-operative cases	Procedure: Osteotomy and interposition of a fascial graft. Postoperative ROM: 41.0°. Functional outcome: Subjective good outcome. Complications: 5.
Bishay SN (2016) [29]	Case series	12 patients / 14 forearms	CRUS; 12 surgical and 0 non-operative cases	Procedure: Osteotomy of the radius and ulna. Forearm position: 70.7° pronation preoperatively to 25.0° pronation postoperatively. Functional outcome: Subjective improvement. Complications: 0.
Hwang JH et al. (2015) [30]	Case series	25 patients / 28 forearms	CRUS; 25 surgical and 0 non-operative cases	Procedure: Osteotomy of the radius and ulna. Forearm position: 47.0° pronation preoperatively to 27.0° supination postoperatively. Functional outcome: Liverpool Elbow Score. Complications: 0.
Simcock X et al. (2015) [31]	Case series	26 patients / 31 forearms	CRUS; 26 surgical and 0 non-operative cases	Procedure: Synostosis osteotomy. Forearm position: 85.0° pronation preoperatively to 8.0° pronation postoperatively. Functional outcome: Not reported. Complications: 3.
Sakamoto S et al. (2014) [32]	Case series	14 patients / 17 forearms	CRUS; 14 surgical and 0 non-operative cases	Procedure: Osteotomy and interposition of a fascial graft. Postoperative ROM: 32°-80° (varied by CRUS subtype). Functional outcome: Not reported. Complications: 0.
Shingade VU et al. (2014) [33]	Case series	28 patients / 30 forearms	CRUS; 28 surgical and 0 non-operative cases	Procedure: Osteotomy of the radius and ulna. Forearm position: 56.0° pronation preoperatively to 27.0° supination postoperatively. Functional outcome: Jebsen-Taylor. Complications: 0.
Horii E et al. (2014) [34]	Case series	26 patients / 35 forearms	CRUS; 26 surgical and 0 non-operative cases	Procedure: Mid-shaft radial osteotomy. Forearm position: 72.0° pronation preoperatively to 0.0° postoperatively. Functional outcome: Subjective improvement. Complications: 3.
Rubin G et al. (2013) [35]	Case series	4 patients / 7 forearms	CRUS; 4 surgical and 0 non-operative cases	Procedure: Synostosis osteotomy and gradual correction with a frame. Forearm position: 100.0° pronation preoperatively to 15° supination postoperatively. Functional outcome: Subjective improvement. Complications: 2.
Burnei G et al. (2013) [36]	Case series	2 patients / 2 forearms	CRUS; 2 surgical and 0 non-operative cases	Procedure: Osteotomy and interposition of a prosthesis. Postoperative ROM: 67.0°. Functional outcome: Jebsen-Taylor and Failla. Complications: 0.
Chen CL et al. (2012) [37]	Case series	2 patients / 2 forearms	CRUS; 2 surgical and 0 non-operative cases	Procedure: Osteotomy and interposition of a fascial graft. Postoperative ROM: 67.5°. Functional outcome: Subjective improvement. Complications: 0.
Ezaki M and Oishi SN (2012) [38]	Case report	1 patient / 1 forearm	CRUS; 1 surgical and 0 non-operative cases	Procedure: Osteotomy of the radius and ulna. Forearm position: Not reported. Functional outcome: Not reported. Complications: Not reported.
Avadhani​ R et al. (2011) [7]	Case report	1 patient / 2 forearms	CRUS; 0 surgical and 1 non-operative case	Procedure: Non-operative. Forearm position: Not reported. Functional outcome: Not reported. Complications: 0.
Siemianowicz A et al. (2010) [39]	Case report	1 patient / 1 forearm	CRUS; 1 surgical and 0 non-operative cases	Procedure: Osteotomy of the radius. Forearm position: Not reported. Functional outcome: Subjective improvement. Complications: Not reported.
Hung NN (2008) [40]	Case series	34 patients / 52 forearms	CRUS; 34 surgical and 0 non-operative cases	Procedure: Osteotomy of the radius and ulna. Forearm position: 82.0-74.0° pronation preoperatively to 6.0-10.0° pronation postoperatively. Functional outcome: Subjective improvement. Complications: 5.
El-Adl W (2007) [41]	Case series	9 patients / 11 forearms	CRUS; 9 surgical and 0 non-operative cases	Procedure: Two-stage osteotomy of the radius and ulna. Forearm position: 76.0° pronation preoperatively to 11.0° pronation postoperatively. Functional outcome: Subjective improvement. Complications: 0.
Dalton JF 4th et al. (2006) [42]	Case series	24 patients / 24 forearms	CRUS; 24 surgical and 0 non-operative cases	Procedure: Osteotomy of the ulna and radius. Forearm position: Not reported. Functional outcome: Not reported. Complications: 3.
Ramachandran M et al. (2005) [3]	Case series	5 patients / 6 forearms	CRUS; 5 surgical and 0 non-operative cases	Procedure: Osteotomy of the radius and ulna. Forearm position: 68.0° pronation preoperatively to 10.0° supination postoperatively. Functional outcome: Subjective improvement. Complications: 1.
Fujimoto M et al. (2005) [43]	Case series	3 patients / 4 forearms	CRUS; 3 surgical and 0 non-operative cases	Procedure: Osteotomy of the radius. Forearm position: 76.0° pronation preoperatively to 2.0° pronation postoperatively. Functional outcome: Subjective improvement. Complications: 1.
Kao HK et al. (2005) [44]	Case report	1 patient / 2 forearms	CRUS; 1 surgical and 0 non-operative cases	Procedure: Osteotomy and interposition of a groin flap. Postoperative ROM: 120.0°. Functional outcome: Subjective improvement. Complications: 0.
Karatosun V et al. (2004) [45]	Case report	1 patient / 2 forearms	CRUS; 1 surgical and 0 non-operative cases	Procedure: Resection of the radius. Postoperative ROM: 140.0°. Functional outcome: Subjective improvement. Complications: 0.
Funakoshi T et al. (2004) [46]	Case report	1 patient / 2 forearms	CRUS; 1 surgical and 0 non-operative cases	Procedure: Osteotomy and interposition of a fascial graft. Postoperative ROM: 70.0°. Functional outcome: Not reported. Complications: 0.
Murase T et al. (2003) [4]	Case series	4 patients / 5 forearms	CRUS; 4 surgical and 0 non-operative cases	Procedure: Osteotomy of the radius and ulna. Forearm position: 78.0° pronation preoperatively to 7.5° pronation postoperatively. Functional outcome: Subjective improvement. Complications: 1.
Lescault E et al. (2000) [47]	Case report	1 patient / 2 forearms	CRUS; 0 surgical and 1 non-operative case	Procedure: Non-operative. Forearm position: Not reported. Functional outcome: Not reported. Complications: 0.
Kanaya F and Ibaraki K (1998) [48]	Case series	7 patients / 7 forearms	CRUS; 7 surgical and 0 non-operative cases	Procedure: Osteotomy and interposition of a fascial graft. Postoperative ROM: 71.0°. Functional outcome: Subjective improvement. Complications: 0.
Guma M and Teitel AD (1996) [49]	Case series	3 patients / 3 forearms	CRUS; 1 surgical and 2 non-operative cases	Procedure: Interposition of an anconeus graft. Postoperative ROM: 150°. Functional outcome: Subjective improvement. Complications: 0.
Lin HH et al. (1995) [50]	Case series	10 patients / 12 forearms	CRUS; 10 surgical and 0 non-operative cases	Procedure: Not reported. Forearm position: 56.0° pronation preoperatively to 8.3° pronation postoperatively. Functional outcome: Not reported. Complications: 0.
Bolano LE (1994) [51]	Case report	1 patient / 1 forearm	CRUS; 1 surgical and 0 non-operative cases	Procedure: Synostosis osteotomy and gradual correction with a frame. Forearm position: 150.0° pronation preoperatively to 0° postoperatively. Functional outcome: Subjective improvement. Complications: 1.
Khalil I and Vizkelety T (1993) [52]	Case series	10 patients / 16 forearms	CRUS; 8 surgical and 2 non-operative cases	Procedure: Synostosis osteotomy. Forearm position: 52.0° pronation preoperatively to 10.0° pronation postoperatively. Functional outcome: Subjective good outcome. Complications: 0.
Bauer M and Jonsson K(1988) [53]	Case series	3 patients / 4 forearms	CRUS; 1 surgical and 2 non-operative cases	Procedure: Osteotomy and interposition of a fat graft. Postoperative ROM: 0°. Functional outcome: Subjective improvement. Complications: 1.
Ogino T and Hikino K (1987) [54]	Case series	40 patients / 55 forearms	CRUS; 11 surgical and 29 non-operative cases	Procedure: Synostosis osteotomy. Forearm position: 65.8° pronation preoperatively to 4.2° supination postoperatively. Functional outcome: Subjective improvement. Complications: 2.
Hankin FM et al. (1987) [55]	Case series	2 patients / 2 forearms	CRUS; 2 surgical and 0 non-operative cases	Procedure: Synostosis osteotomy. Forearm position: 65.0° pronation preoperatively to 20.0° pronation postoperatively. Functional outcome: Not reported. Complications: 2.
Cleary JE and Omer GE (1985) [1]	Case series	27 patients / 41 forearms	CRUS; 4 surgical and 23 non-operative cases	Procedure: Osteotomy (varied techniques). Forearm position: Not reported. Functional outcome: Jebsen-Taylor. Complications: 2.
Miura T et al. (1984) [56]	Case series	7 patients / 8 forearms	CRUS; 7 surgical and 0 non-operative cases	Procedure: Synostosis osteotomy and interposition of the anconeus. Postoperative ROM: 0°. Functional outcome: Subjective improvement. Complications: 1.
Simmons BP et al. (1983) [5]	Case series	20 patients / 32 forearms	CRUS; 20 surgical and 0 non-operative cases	Procedure: Osteotomy (various techniques). Forearm position: 82.0° pronation preoperatively to 8.0° pronation postoperatively. Functional outcome: Subjective improvement. Complications: 8.
Danielsson LG (1980) [57]	Case report	1 patient / 1 forearm	CRUS; 1 surgical and 0 non-operative cases	Procedure: Synostosis osteotomy. Forearm position: Not reported. Functional outcome: Not reported. Complications: 1.
Green WT and Mital MA (1979) [58]	Case series	15 patients / 25 forearms	CRUS; 13 surgical and 2 non-operative cases	Procedure: Synostosis osteotomy. Forearm position: 76.0° pronation preoperatively to 5.0° supination postoperatively. Functional outcome: Subjective improvement. Complications: 2.
Hansen OH and Andersen NO (1970) [6]	Case series	2 patients / 2 forearms	CRUS; 1 surgical and 0 non-operative cases	Procedure: Osteotomy of the radius. Forearm position: Not reported. Functional outcome: Subjective little change. Complications: 1.

Using the JBI critical appraisal tools, risk of bias was rated as low in 30/55 studies (54.5%), moderate in 22/55 (40.0%), and high in 3/55 (5.5%). Full details of the risk of bias analysis are presented in Table 2 and Table 3.

**Table 2:** JBI critical appraisal checklist for the included case series. JBI: Joanna Briggs Institute

Reference (author, year)	Q1 Inclusion criteria clearly defined?	Q2 Condition measured reliably for all participants?	Q3 Valid method used for identifying the condition?	Q4 Consecutive inclusion of participants?	Q5 Complete inclusion of participants?	Q6 Participant demographics clearly reported?	Q7 Clinical information clearly reported?	Q8 Outcomes clearly reported?	Q9 Site demographics clearly described?	Q10 Appropriate statistical analysis?	Yes (n)	Overall risk of bias
Zarantonello P et al. (2025) [11]	Yes	Yes	Yes	Yes	Yes	Yes	Yes	Yes	Yes	Yes	10	Low
Dong Y et al. (2024) [12]	Yes	Yes	Yes	Yes	Yes	Yes	Yes	Yes	Yes	Yes	10	Low
Luo X et al. (2024) [13]	Yes	Yes	Yes	Yes	Yes	Yes	Yes	Yes	Yes	Yes	10	Low
Kanaya F et al. (2023) [14]	Yes	Yes	Yes	Yes	Yes	Yes	Yes	Yes	Yes	Yes	10	Low
Gandhi S et al. (2023) [15]	Yes	Yes	Yes	Yes	Yes	Yes	No	No	No	N/A	6	Moderate
Bo H et al. (2023) [16]	No	Yes	Yes	No	No	No	No	No	Yes	No	3	High
Tan W et al. (2022) [17]	Yes	Yes	Yes	No	No	Yes	Yes	Yes	Yes	Yes	8	Low
Martinez-Alvarez S et al. (2022) [18]	Yes	Yes	Yes	No	No	Yes	Yes	Yes	Yes	N/A	7	Moderate
Bai F et al. (2022) [19]	Yes	Yes	Yes	No	No	Yes	Yes	Yes	Yes	N/A	7	Moderate
Hamiti Y et al. (2022) [20]	Yes	Yes	Yes	No	No	Yes	Yes	Yes	Yes	Yes	8	Low
Zhang ZQ et al. (2021) [21]	Yes	Yes	Yes	No	No	Yes	Yes	Yes	Yes	N/A	7	Moderate
Pei X and Han J (2019) [24]	Yes	Yes	Yes	Yes	Yes	Yes	Yes	Yes	Yes	N/A	9	Low
Satake H et al. (2018) [26]	Yes	Yes	Yes	Yes	Yes	Yes	Yes	Yes	No	N/A	8	Low
Kanaya K et al. (2016) [28]	Yes	Yes	Yes	Yes	Yes	Yes	Yes	Yes	Yes	N/A	9	Low
Bishay SN (2016) [29]	Yes	Yes	Yes	Yes	Yes	Yes	Yes	Yes	No	Yes	9	Low
Hwang JH et al. (2015) [30]	Yes	Yes	Yes	No	No	Yes	Yes	Yes	No	Yes	7	Moderate
Simcock X et al. (2015) [31]	Yes	Yes	Yes	Yes	Yes	Yes	Yes	Yes	No	N/A	8	Low
Sakamoto S et al. (2014) [32]	Yes	Yes	Yes	Yes	Yes	Yes	Yes	Yes	No	N/A	8	Low
Shingade VU et al. (2014) [33]	Yes	Yes	Yes	Yes	Yes	Yes	Yes	Yes	Yes	Yes	10	Low
Horii E et al. (2014) [34]	Yes	Yes	Yes	No	No	Yes	Yes	Yes	No	N/A	6	Moderate
Rubin G et al. (2013) [35]	No	Yes	Yes	No	No	Yes	Yes	Yes	No	N/A	5	Moderate
Burnei G et al. (2013) [36]	Yes	Yes	Yes	Yes	Yes	No	No	Yes	No	N/A	6	Moderate
Chen CL et al. (2012) [37]	No	Yes	Yes	No	No	No	Yes	Yes	No	N/A	4	High
Hung NN (2008) [40]	Yes	Yes	Yes	Yes	Yes	Yes	Yes	Yes	No	N/A	8	Low
El-Adl W (2007) [41]	Yes	Yes	Yes	Yes	Yes	Yes	Yes	Yes	No	N/A	8	Low
Dalton JF 4th et al. (2006) [42]	Yes	Yes	Yes	Yes	Yes	Yes	Yes	Yes	Yes	Yes	10	Low
Ramachandran M et al. (2005) [3]	No	Yes	Yes	No	No	Yes	Yes	Yes	No	N/A	5	Moderate
Fujimoto M et al. (2005) [43]	Yes	Yes	Yes	Yes	Yes	Yes	Yes	Yes	No	N/A	8	Low
Murase T et al. (2003) [4]	No	Yes	Yes	No	No	Yes	Yes	Yes	No	N/A	5	Moderate
Kanaya F and Ibaraki K (1998) [48]	No	Yes	Yes	No	No	Yes	Yes	Yes	No	N/A	5	Moderate
Guma M and Teitel AD (1996) [49]	No	Yes	Yes	No	No	No	Yes	Yes	No	N/A	4	High
Lin HH et al. (1995) [50]	Yes	Yes	Yes	No	No	No	Yes	Yes	No	N/A	5	Moderate
Khalil I and Vizkelety T (1993) [52]	Yes	Yes	Yes	No	No	Yes	Yes	Yes	No	N/A	6	Moderate
Bauer M and Jonsson K (1988) [53]	No	Yes	Yes	No	No	Yes	Yes	Yes	No	N/A	5	Moderate
Ogino T and Hikino K (1987) [54]	No	Yes	Yes	No	No	Yes	Yes	Yes	Yes	N/A	6	Moderate
Hankin FM et al. (1987) [55]	No	Yes	Yes	No	No	Yes	Yes	Yes	No	N/A	5	Moderate
Cleary JE and Omer GE (1985) [1]	Yes	Yes	Yes	Yes	Yes	Yes	Yes	Yes	Yes	N/A	9	Low
Miura T et al. (1984) [56]	No	Yes	Yes	No	No	Yes	Yes	Yes	Yes	N/A	6	Moderate
Simmons BP et al. (1983) [5]	Yes	Yes	Yes	Yes	Yes	Yes	Yes	Yes	Yes	N/A	9	Low
Green WT and Mital MA (1979) [58]	No	Yes	Yes	No	No	Yes	Yes	Yes	No	N/A	5	Moderate
Hansen OH et al. (1970) [6]	Yes	Yes	Yes	Yes	Yes	No	No	No	Yes	N/A	6	Moderate

**Table 3:** JBI critical appraisal checklist for the included case reports. JBI: Joanna Briggs Institute.

Reference (author, year)	Q1 Patient demographics clearly described?	Q2 Patient history clearly described as a timeline?	Q3 Clinical condition on presentation clearly described?	Q4 Diagnostic tests/assessment methods described?	Q5 Intervention(s) described?	Q6 Post-intervention outcomes described?	Q7 Adverse events described?	Q8 Takeaway lessons provided?	Yes (n)	Overall risk of bias
Kepenek-Varol B and Hoşbay Z (2020) [22]	Yes	Yes	Yes	Yes	Yes	Yes	Yes	Yes	8	Low
Jia Y et al. (2020) [23]	Yes	Yes	Yes	Yes	Yes	Yes	Yes	Yes	8	Low
Barrera-Ochoa S et al. (2019) [25]	Yes	Yes	Yes	Yes	Yes	Yes	Yes	Yes	8	Low
Natwa N et al. (2018) [8]	Yes	Yes	Yes	Yes	Yes	Yes	Yes	Yes	8	Low
Tsai J (2017) [27]	No	Yes	Yes	Yes	Yes	Yes	Yes	Yes	7	Low
Ezaki M and Oishi SN (2012) [38]	No	No	Yes	Yes	Yes	No	No	Yes	4	Moderate
Avadhani​ R et al. (2011) [7]	Yes	Yes	Yes	Yes	No	No	No	Yes	5	Moderate
Siemianowicz A et al. (2010) [39]	Yes	Yes	Yes	Yes	Yes	No	No	Yes	6	Low
Kao HK et al. (2005) [44]	Yes	Yes	Yes	Yes	Yes	Yes	Yes	Yes	8	Low
Karatosun V et al. (2004) [45]	No	Yes	Yes	Yes	Yes	Yes	No	Yes	6	Low
Funakoshi T et al. (2004) [46]	No	Yes	Yes	Yes	Yes	Yes	No	Yes	6	Low
Lescault E et al. (2000) [47]	Yes	Yes	Yes	No	No	No	No	Yes	4	Moderate
Bolano LE (1994) [51]	No	No	Yes	Yes	Yes	Yes	Yes	Yes	6	Low
Danielsson LG (1980) [57]	No	Yes	Yes	Yes	Yes	Yes	Yes	Yes	7	Low

Among case series (n = 41), 19 (46.3%) were judged to be at low risk of bias, 19 (46.3%) at moderate risk, and 3 (7.3%) at high risk. The most common limitations were poor reporting of site demographics/setting (Q9; 19/41, 46.3%) and lack of consecutive and complete participant inclusion (Q4 and Q5; 20/41, 48.8% each). Condition measurement and diagnosis were adequate in all studies (Q2 and Q3; 100%). Outcomes (Q8; 38/41, 92.7%), clinical information (Q7; 37/41, 90.2%), and participant demographics (Q6; 35/41, 85.4%) were generally well reported. Statistical analysis was infrequently performed (Q10; 8/41, 19.5%), reflecting the predominance of small, descriptive case series.

Among case reports (n = 14), 11 (78.6%) were rated as low risk of bias and 3 (21.4%) as moderate risk. No case reports were rated as high risk. Case reports consistently described the clinical condition, diagnostic assessment, interventions, outcomes, and key takeaway lessons (Q3, Q8; 100%). The most common limitations were incomplete reporting of patient demographics (Q1; 6/14, 42.9%) and lack of clarity on reporting harms or adverse events (Q7; 6/14, 42.9%).

Indications for Surgery

Indications for surgical intervention were reported in 17 papers. Subjective functional limitations were given as the sole indication in 11 papers [3,4,6,15,30,44,48,50,52,54,58]. Other papers used a variety of objective measures, such as the Failla Classification [59], Jebsen-Taylor Hand Function Test [60], QuickDASH [61], Liverpool Elbow Score [62], or their own objective measure of ability to perform ADLs, as indications for surgical management. Four papers reported good functional ability as a reason to avoid surgery in 13 patients [34,49,52,58].

A fixed pronation deformity of 60˚ or more was given as an absolute indication for surgery in three papers [5,17,18]. Ramachandran M et al. described 68˚ of fixed pronation as causing ‘significant disability’ and used this as an indication for surgery [3]. Horii E et al. described “severe pronation” deformity as an indication for surgery, but did not define what constituted severe pronation [34]. Pei X and Han J gave a pronation deformity greater than 55˚ and a Failla score less than 10 as inclusion criteria but did not specify the indications for surgery; only 31 of 44 patients undergoing derotation osteotomy for CRUS during the study period were included [24].

Forearm Position

Conservatively managed patients were reported in 12 papers, with a total of 110 reported cases. Forearm position was reported as a mean of 16.8˚ of pronation (range: 90˚ pronation to 20˚ supination) [1,7,8,11,22,27,47,49,52-54,58]. Patients managed surgically were reported in 50 papers. Preoperative forearm position, where reported, was a mean of 63.1˚ (range: 150˚ pronation to 70˚ supination) [4,5,11-20,24,30,34,46,49,53,54]. For patients undergoing surgical procedures resulting in a fixed forearm position, postoperative position was a mean of 1˚ of pronation (range: 38˚ pronation to 27˚ supination) [3-6,11,13,15,17-21,24,26,30,33-35,43,51-55].

Functional Outcomes

Functional assessment was reported in 40 papers. Formal objective scoring systems were used in 11 [1,11-13,15,17,20,24,26,30,33], while the remainder relied on subjective assessment. Zarantonello P et al. were the only authors to directly compare functional outcomes between operative and non-operative groups at final follow-up. In 52 surgically managed and 45 conservatively managed cases, QuickDASH scores were similar between groups (18.9 vs 18.2; p = 0.707), although baseline functional status was not reported and the operative group had significantly greater pre-treatment pronation deformity (69.1° vs 15.1°; p = 0.0001) [11]. Cleary and Omer, by contrast, suggested that the degree of fixed forearm deformity does not necessarily predict functional limitation, finding no clear association between forearm position and performance on the Jebsen-Taylor test in a non-operatively managed cohort [1].

The Failla classification [59], an objective measure of forearm function, was used in seven papers to assess functional outcomes after operative intervention [12,13,15,17,20,24,33]. Collectively, these studies demonstrated improved postoperative forearm function following derotational osteotomy or related procedures. Pei X and Han J reported a shift from predominantly fair or poor preoperative grades to 34 excellent and two good postoperative outcomes [24], while Shingade VU et al. found significant improvement in both Failla and Jebsen-Taylor scores [33]. Similar postoperative improvement was reported by Dong Y et al., Gandhi S et al., Tan w et al., and Hamiti Y et al., although baseline Failla scores were inconsistently reported [12,15,17,20].

Other objective functional outcome measures were also used. Hwang JH et al. used the Liverpool Elbow Score to evaluate function before and after derotational osteotomy in 25 patients. They reported a statistically significant improvement in mean score from 36 to 42 points [30].

Procedures to Restore Range of Motion

Procedures intended to restore forearm rotation through excision of the synostosis have been described using a variety of techniques, with variable success. Simple synostosis resection, including cases performed without interposition or with adipose or fascial graft interposition, was associated with recurrence and failure to maintain lasting motion [6,23,36,53]. However, despite synostosis recurrence, functional ability was reported to improve subjectively in some patients due to the forearms being fixed in a more neutral position [53].

More favourable outcomes were reported following synostosis resection combined with free flap interposition. The largest series were reported by Kanaya F et al., who described sustained postoperative rotation following resection and free vascularised fascio-fat graft interposition, although preoperative anterior radial head dislocation and severe pronation ankylosis were associated with poorer outcomes [14,28,48].

Similar preservation of motion and low recurrence were described with several modified techniques, including local vascularised fat grafts, groin and thigh flaps, latissimus dorsi free flaps, and local tissue transfer [12,19,32,37,44,46,49]. Additional isolated reports described successful outcomes following incomplete synostosis resection without interposition [45], and more complex reconstructive strategies using prosthetic or osseous replacement [25,36].

Among studies reporting postoperative motion following reconstructive procedures, range of motion was described in 75 patients, with a mean arc of 75° [12,14,19,28,36,37,44,46,48,49,53,63].

Complications

There were a total of 61 reported complications, all of which occurred in those undergoing surgical management. This accounts for 11.1% of surgically managed patients. Reported complications included nerve palsies in 20 patients (3.6%) [6,11,19,24,28,35,48,54-57], recurrence in 10 patients (1.8%) [6,19,23,36,53], loss of alignment in eight patients (1.4%) [1,4,5,19,34], vascular compromise in five patients (0.9%) [1,5,58], malunion/non-union/delayed union in five patients (0.9%) [1,17,43,56], infection in five patients (0.9%) [5,12,34,35,53], and compartment syndrome in four patients (0.7%) [11,24,58]. The remaining 4 complications were residual radial head dislocation in two patients (0.4%), flap congestion in one patient (0.2%), and a synovial fold causing elbow locking in one patient (0.2%). All of these were reported by Kanaya K et al. 2016 [28].

Although overall complication rates were similar between single-stage and staged derotational procedures (12.6% vs 12.3%), serious complications such as ischaemia and compartment syndrome were reported only following single-stage correction. Single-stage derotation, performed at the time of osteotomy, was reported in 438 patients across 37 studies. Complications occurred in 55 patients (12.6%), including 20 nerve palsies, loss of alignment in seven, ischaemic complications in five, recurrence in three, and compartment syndrome in four. Additional complications included wound infection, delayed union, and persistent radial head dislocation [1,3-6,11,12,17,19,20,23-26,30,31,33-37,43-46,48,49,52-57,63].

Staged or gradual derotation using external fixation was reported in 81 patients across nine studies. Complications occurred in 10 cases (12.3%). The most serious reported event was a transient neuropraxia following acute correction, which resolved after the degree of correction was reduced; the patient subsequently underwent gradual correction using an Ilizarov frame. Other reported complications included pin-site infection and delayed union [16,18,35,38,39,41,42,50,51].

Discussion 

The decision to offer surgical management for CRUS remains controversial, as there is no universally accepted indication for intervention. In most studies, surgery is reserved for patients with substantial functional limitation, assessed using both objective scoring systems and subjective patient- or caregiver-reported measures. This patient-specific approach appears justified given the relatively high complication rates associated with operative treatment. Severity of pronation deformity, most commonly >60°, has also been proposed as an indication for surgery. In addition, bilateral involvement is frequently reported as a relative indication. This is supported by the literature, with operatively managed patients demonstrating a higher rate of bilateral disease (29.4% vs 4.6%) and a greater mean fixed pronation deformity (63.1° vs 16.8°) than those managed non-operatively. However, these factors should not be considered in isolation. Patients with marked pronation deformity and/or bilateral disease have been reported to retain good function [1].

The literature describes two main surgical approaches to CRUS. The first is derotational osteotomy, which repositions the forearm into a more functional fixed position by reducing the pronation deformity. Although this approach does not restore active forearm rotation, it generally provides reliable correction of forearm position. Single-stage correction appears to be associated with a higher risk of serious complications than staged procedures, particularly in patients requiring large angular corrections. Staged procedures may therefore be preferable in selected cases.

The second approach aims to restore forearm rotation through resection of the synostosis and interposition of graft material. This technique has the potential to improve both resting forearm position and rotational movement. However, it is technically more demanding, has shown variable outcomes, and appears to be associated with a relatively high rate of recurrence. In addition, most successful reports originate from a single centre and surgeon, Kanaya, which limits confidence in the generalisability and reproducibility of these results. This is particularly relevant because the preoperative pronation deformity reported in Kanaya’s series was a low outlier compared with the wider literature (mean 31.9° vs 63.1°), and the authors themselves reported worse outcomes in patients with greater preoperative pronation deformity [14,28,48].

Target positions of derotation in the literature vary, ranging from slight pronation in the dominant forearm to slight supination in the non-dominant forearm, reflecting the absence of consensus on an ideal postoperative alignment [4,7,51,55,59]. Favourable functional outcomes have been reported across a range of final forearm positions, suggesting that the key determinant of outcome is not attainment of a specific angle, but correction of severe fixed pronation towards a more neutral resting position. This interpretation is supported by Zarantonello P et al., who reported the largest comparative series in the literature, including 52 operative and 45 non-operative cases. Although the surgically managed group had substantially greater pretreatment pronation deformity, functional outcomes at final follow-up were similar between groups, suggesting that derotational surgery may restore function to a level comparable with that seen in less severely affected patients [11]. The aim of derotational surgery should be to achieve the most functional near-neutral position possible for the individual patient, while balancing the constraints of soft tissues and the greater complication risk associated with large angular corrections.

Timing of surgery was not a primary outcome of this review. Nevertheless, limited evidence suggests that earlier intervention may be associated with improved outcomes. Dalton JF 4th et al. reported a higher non-union rate in older patients undergoing rotational osteotomy for fixed forearm deformities, including CRUS [42]. In addition, Luo X et al. reported superior Failla scores at follow-up in patients undergoing derotational osteotomy before the age of 8 years compared with those treated later (14.3 vs 13.0) [13]. These findings suggest there may be benefit in proceeding to surgery early if indicated.

Little work has been done on the impact of formal physical therapy on CRUS outcomes as an alternative to operative intervention. A single paper reported short-term hand therapy as positively affecting functional measures, but not change in ROM [22]. Further work to assess the role of physiotherapy would be beneficial, particularly its impact on older patients given the inferior surgical outcomes in this group.

The findings of this review should be interpreted in the context of several limitations, largely due to the available literature. Included studies were case series and case reports, largely without comparative designs. This limits causal inference and means outcomes are vulnerable to selection bias. As a result, comparisons between operative and non-operative cohorts are limited. There was also substantial heterogeneity in surgical techniques, postoperative protocols, and goals of surgery. Outcomes were reported inconsistently, ranging from resting forearm position to arcs of rotation and a mixture of subjective and objective functional measures. This variability limited meaningful quantitative analysis. Reporting quality was variable across studies. The risk of bias assessment identified frequent limitations in reporting of patient selection and setting/site characteristics. This restricts generalisability and may inflate perceived benefit if favourable outcomes are preferentially reported. Follow-up was inconsistently reported, making long-term outcomes hard to quantify. Complications were also inconsistently defined and reported. Finally, the rarity of CRUS and the predominance of single-centre retrospective series increase the risk of publication bias.

Future research would benefit from multicentre collaboration with standardised reporting. Even where randomised trials are not feasible, well-designed comparative observational studies with adjustment for baseline severity would substantially strengthen the evidence base.

## Conclusions

Given the limited availability of high-quality studies, it remains challenging to draw definitive conclusions regarding the management of CRUS. Operative treatment may improve function, primarily by correcting severe fixed pronation to a more functional, near-neutral position. However, it carries a meaningful risk of complications. In addition, patients with significant deformity have been reported to have good function without intervention. Management should therefore be individualised according to functional limitation, compensatory shoulder and elbow motion, deformity severity, and bilateral involvement. Further high-quality research in this area is required to reach a consensus on the optimal management of this rare condition.
